# Protection from illegal fishing and shark recovery restructures mesopredatory fish communities on a coral reef

**DOI:** 10.1002/ece3.5575

**Published:** 2019-08-20

**Authors:** Conrad W. Speed, Matthew J. Rees, Katherine Cure, Brigit Vaughan, Mark G. Meekan

**Affiliations:** ^1^ Australian Institute of Marine Science Indian Ocean Marine Research Centre UWA (MO96) Crawley WA Australia; ^2^ Global FinPrint Project Indian Ocean Marine Research Centre UWA (MO96) Crawley WA Australia

**Keywords:** baited remote underwater video stations, competition, elasmobranchs, fishing pressure, marine reserve, predation, top‐down effects

## Abstract

The recovery of communities of predatory fishes within a no‐take marine reserve after the eradication of illegal fishing provides an opportunity to examine the role of sharks and other large‐bodied mesopredatory fishes in structuring reef fish communities. We used baited remote underwater video stations to investigate whether an increase in sharks was associated with a change in structure of the mesopredatory fish community at Ashmore Reef, Western Australia. We found an almost fourfold increase in shark abundance in reef habitat from 0.64 hr^−1^ ± 0.15 *SE* in 2004, when Ashmore Reef was being fished illegally, to 2.45 hr^−1^ ± 0.37 in 2016, after eight years of full‐time enforcement of the reserve. Shark recovery in reef habitat was accompanied by a two and a half‐fold decline in the abundance of small mesopredatory fishes (≤50 cm TL) (14.00 hr^−1^ ± 3.79 to 5.6 hr^−1^ ± 1.20) and a concomitant increase in large mesopredatory fishes (≥100 cm TL) from 1.82 hr^−1^ ± 0.48 to 4.27 hr^−1^ ± 0.93. In contrast, near‐reef habitats showed an increase in abundance of large mesopredatory fishes between years (2.00 hr^−1^ ± 0.65 to 4.56 hr^−1^ ± 1.11), although only smaller increases in sharks (0.67 hr^−1^ ± 0.25 to 1.22 hr^−1^ ± 0.34) and smaller mesopredatory fishes. Although the abundance of most mesopredatory groups increased with recovery from fishing, we suggest that the large decline of small mesopredatory fish in reef habitat was mostly due to higher predation pressure following the increase in sharks and large mesopredatory fishes. At the regional scale, the structure of fished communities at Ashmore Reef in 2004 resembled those of present day Scott Reefs, where fishing still continues today. In 2016, Ashmore fish communities resembled those of the Rowley Shoals, which have been protected from fishing for decades.

## Graphical abstract

We observed abundance and community composition of shark and mesopredatory reef fish at Ashmore Reef in Western Australia, before and after recovery from illegal fishing pressure. After eight years of recovery, there was a large decrease in small (≤50 cm TL) mesopredatory reef fish abundance and a concomitant increase in shark and large (>100 cm TL) mesopredatory fish abundance in reef habitat. Our findings provide support for fish community restructuring with reef shark recovery, which has implications for management time frames of marine protected areas.

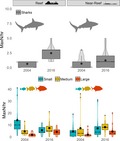

## 1 INTRODUCTION

Large predators can structure ecosystems in terrestrial and aquatic environments (Estes et al., 2011) through both consumption of prey and by influencing prey distribution and behavior (Creel & Christianson, 2008; Heithaus, Wirsing, Burkholder, Thomson, & Dill, 2009; Ripple & Beschta, 2004). These roles are mediated by habitat complexity and the community structure of the predator guild (Ritchie & Johnson, 2009). In some marine environments, notably coral reefs, evidence for the role of large predators in the top‐down regulation of ecosystems remains contentious (Casey et al., 2017), although studies have indicated that important processes such as herbivory can be suppressed around reefs through the fear of predation (Madin, Madin, & Booth, 2011; Rizzari, Frisch, Hoey, & McCormick, 2014). Manipulative experiments in these environments that might resolve this issue by altering abundances of sharks and other large teleost predators (e.g., serranids and carangids) pose logistic, financial, and ethical difficulties (Baum & Worm, 2009), given that these animals are large‐bodied, reproduce slowly (Cortes, 2000), tend to occur in low numbers (Nadon et al., 2012), and move over relatively large areas (often entire reefs; Heupel, Knip, Simpfendorfer, & Dulvy, 2014). Consequently, some researchers have used a comparative approach, examining the structure of communities of fishes on reefs where sharks have been reduced in numbers by fishing with those of protected reefs where shark populations remain largely intact (Barley, Meekan, & Meeuwig, 2017b; Ruppert, Travers, Smith, Fortin, & Meekan, 2013; Sandin et al., 2008). In some cases, the results of these comparisons are consistent with expectations from theory and have suggested that the presence of reef sharks influences the abundance, diet, condition, and morphology of mesopredatory fishes (Barley, Meekan, & Meeuwig, 2017a; Hammerschlag et al., 2018), and ultimately may affect the resilience of reef systems to disturbance (Ruppert et al., 2013, although see; Rizzari, Bergseth, & Frisch, 2015). However, as with all comparative studies of this type, other potential explanations exist for many of these patterns, particularly since results are based on observations that have limited spatial replication (single sets of reef systems on adjacent areas of shelf) and may also be confounded by human impacts other than fishing for sharks (Casey et al., 2017). Furthermore, redundancy in functional traits of reef fish communities (Mouillot et al., 2014) has led some researchers to suggest that the loss of sharks may simply result in other species occupying their broad functional role or trophic position in food webs (Ferretti, Worm, Britten, Heithaus, & Lotze, 2010; Frisch et al., 2016; Kitchell, Essington, Boggs, Schindler, & Walters, 2002; Roff et al., 2016a). For these reasons, the importance of reef sharks as structuring agents of communities of coral reef fishes remains largely unresolved (e.g., Roff et al., 2016b; Ruppert, Fortin, & Meekan, 2016).

Comparisons of fish communities through time, rather than space, offer an alternative means to examine the role of reef sharks and other large mesopredatory fishes as potential top‐down regulators of community structure. Such studies avoid many of the confounding effects that may be introduced by comparisons across space, where reefs can vary in oceanographic setting, histories of exploitation, habitat structure, and biogeography (Casey et al., 2017; Valdivia, Cox, & Bruno, 2017). Although many populations of reef sharks are declining (e.g., Graham, Spalding, & Sheppard, 2010; Robbins, Hisano, Connolly, & Choat, 2006; Ward‐Paige, Mora, et al., 2010), in a few circumstances, changes in management strategies or better enforcement of existing regulations have allowed numbers of reef sharks to recover (e.g., Espinoza, Cappo, Heupel, Tobin, & Simpfendorfer, 2014; Speed, Cappo, & Meekan, 2018). These offer a unique opportunity to gain insights into the importance of sharks in reef environments and a means to test predictions generated by spatial comparisons through comparisons of the structure of fish communities prior to and after recovery of shark populations.

Here, we examine links between the recovery of reef sharks and the change in composition and abundance of different size classes of mesopredatory reef fishes at Ashmore Reef, an atoll‐like coral reef on the edge of the continental shelf off the northwest coast of Western Australia. At this locality, the continuous presence of management agencies enforcing a no‐take marine reserve that encompasses the entire reef has resulted in the cessation of illegal fishing and the recovery of reef shark populations to levels comparable with other protected reefs in the region over a period of eight years (Speed et al., 2018). In order to test the predictions of earlier spatial studies (e.g., Barley et al., 2017b; Meekan, Cappo, Carleton, & Marriott, 2006; Ruppert et al., 2013), we compared both the pre‐ and postrecovery of communities of mesopredatory fishes at Ashmore Reef to other nearby reefs in the region that are either currently being fished (the Scott Reefs) and have low numbers of sharks or have been protected for almost three decades (the Rowley Shoals) and have relatively “pristine” predator communities. As habitat also influences reef fish communities (Darling et al., 2017; Fitzpatrick, Harvey, Heyward, Twiggs, & Colquhoun, 2012; Friedlander & Parrish, 1998; McLean et al., 2016; Valdivia et al., 2017) and likely drives predator–prey interactions (Ritchie & Johnson, 2009), we compared changes occurring in communities of mesopredatory fishes through time in both reef and near‐reef environments at Ashmore Reef. Contrasts between these habitats may be particularly important given the growing evidence that reefs sharks and other predatory fish species can consume non‐reef‐based prey (Frisch, Ireland, & Baker, 2014; Frisch et al., 2016; McCauley et al., 2012).

## 2 METHODS

### 2.1 Study sites

#### 2.1.1 Ashmore Reef

**Figure 1:**
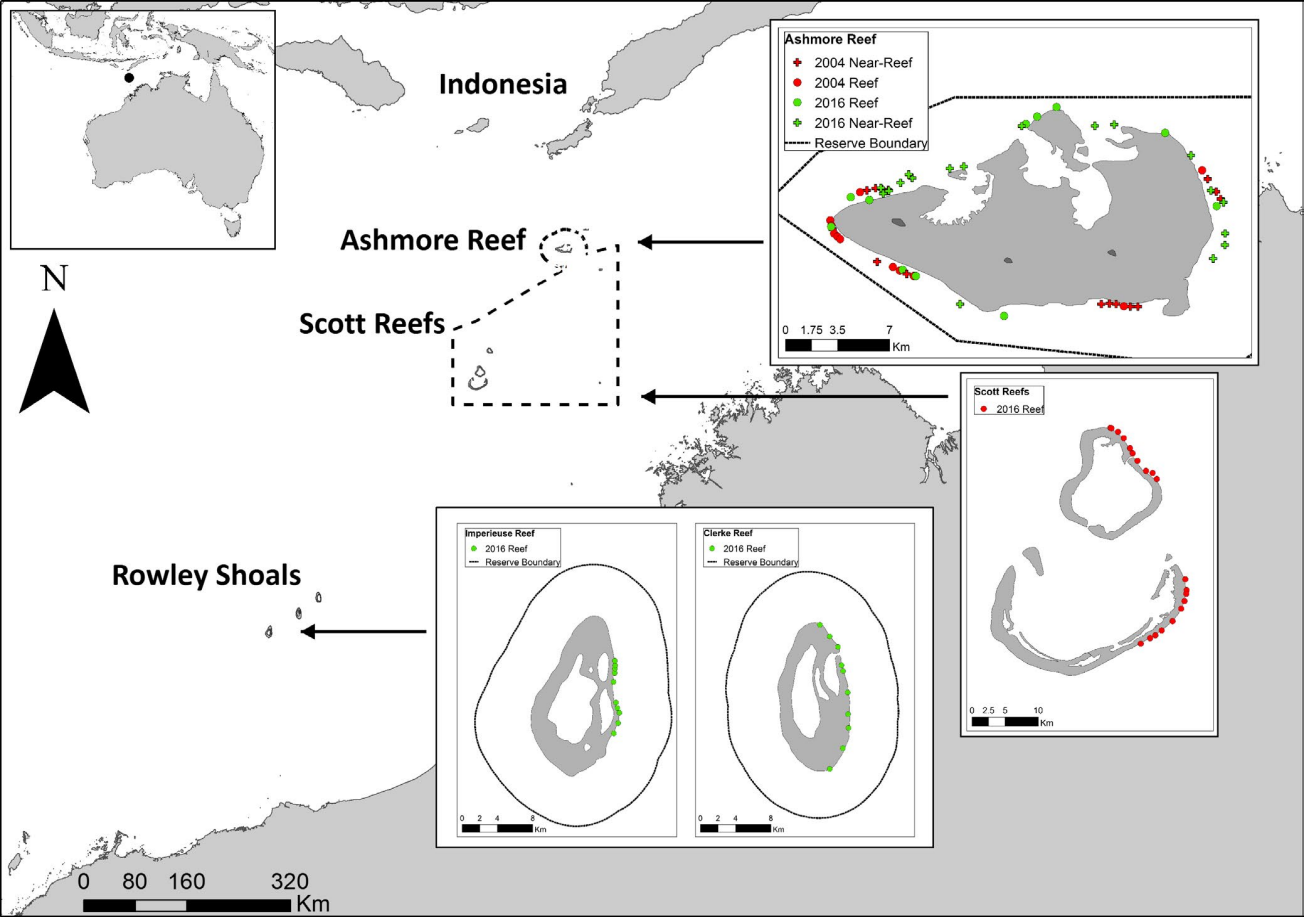
Study sites in northwestern Australia, including the locations of baited remote underwater video stations deployed at Ashmore Reef in 2004 and 2016, and Scott Reefs and Rowley Shoals in 2016 (Imperieuse and Clerke Reefs). The dashed line represents the Memorandum of Understanding Box between Australia and Indonesia. Dotted lines within study site insets represent marine reserve boundaries for Ashmore Reef and Rowley Shoals (Imperieuse and Clerke Reefs)

Ashmore Reef (12°14.929′S, 123°3.319′E) is a platform reef (26 × 14 km) on the North West Shelf of Australia that rises from the edge of the continental slope (Wilson, 2013) (Figure 1). The reef is situated ~350 km from the mainland of Australia and ~145 km to the nearest reef system in Indonesia (Berry, 1993). Ashmore Reef National Nature Reserve was established in 1983 (583 km^2^), although traditional fishing by Indonesian artisanal fishers was permitted through a Memorandum of Understanding (MOU) with the Australian Government until 1988 (Australia, 2002). From this time onwards, a no‐take marine reserve was declared at Ashmore Reef, although a small area within the lagoon was exempted from this restriction to allow subsistence fishing. However, illegal fishing for sharks and reef fishes continued up into the 2000s (Field, Meekan, Buckworth, & Bradshaw, 2009), as enforcement of no‐take regulations was difficult due to the remote location of Ashmore and the close proximity to Indonesia. Australian Border Force vessels made sporadic patrols between 2000 and 2006, and from 2008, a vessel was deployed at Ashmore Reef on a near‐permanent basis (300 continuous days per year) (DIBP, 2017). Due to this enforcement history, Ashmore Reef was considered to be a “fished reef” pre‐2008 and a fully protected reef post‐2008.

### 2.2 Scott Reefs (North & South) and the Rowley Shoals (Imperieuse & Clerke Reefs)

The Scott Reefs consist of three large atolls on the edge of the continental shelf: Seringapatam Reef (8 × 9.4 km); Scott Reef North (16.3 × 14.4 km); and Scott Reef South (27.4 × 17 km), which are ~260 km from the mainland of Australia (14°0′S, 121°45′E) (Figure 1). The Scott Reefs have been fished by Indonesian artisanal fishers who have targeted sharks and some reef fishes since at least the 1800s (Russell & Vail, 1988). The Scott Reefs and Ashmore Reef lie within the “MOU Box” (Figure 1) where Indonesian fishermen are still permitted to fish by agreement with the Australian Government, although not within the Ashmore Reef no‐take marine reserve (Meekan et al., 2006).

The Rowley Shoals are composed of three large reefs: Imperieuse (17.8 × 9.5 km); Clerke (15.8 × 7.6 km); and Mermaid (14.5 × 7.6 km) (Berry, 1986), which are to the south of the MOU Box and share the same position at the edge of the continental shelf as the Scott and Ashmore reefs. Unlike the Scott Reefs, Rowley Shoals are a marine reserve (est. 1990) that is subject to very small amounts of charter fishing focused on pelagic species (billfishes, tunas etc.) (Conservation, 2007; Figure 1). The Rowley Shoals were therefore considered to be a baseline of unfished abundances of sharks and mesopredatory fishes, against which changes in fish community structure at Ashmore Reef could be compared.

A very similar suite of species is present on the offshore reefs of the entire North West Shelf, including Rowley Shoals, Scott Reefs, Ashmore Reef, and Cartier and Hibernia Reefs, and at the level of genus, these communities are similar to other reefs in the tropical Indo‐West Pacific (Russell & Hanley, 1993).

### 2.3 Data collection

Data were collected using baited remote underwater video stations (BRUVS) in shallow water (10–30 m) around Ashmore Reef in both 2004 (*n* = 26) and 2016 (*n* = 29), and the Scott Reefs (*n* = 20) and the Rowley Shoals (*n* = 20) in 2016. All deployments were within 1.5 km from the reef edge to ensure reef‐associated species were the primary focus. The 2004 survey at Ashmore Reef was part of a larger program to assess shark stocks in the MOU Box (Meekan et al., 2006). The 2016 surveys were done as part of the Global FinPrint Project https://globalfinprint.org/. The BRUVS consisted of a galvanized or aluminum frame enclosing a camera housing made from PVC pipe with flat acrylic ports. Sony TRV18E MiniDV Handicams with wide‐angle lenses (0.6×) were used in housings in 2004, and GoPro Hero4 Silver Edition was used in 2016.

A bait bag containing 1 kg of crushed pilchards (*Sardinops* spp.) was suspended at the end of a 1.5 m pole in front of the camera. BRUVS were deployed to provide a minimum of 60 min of video recorded at the seabed. Successive deployments within a set were spaced between 400 and 1,000 m apart in shallow depths (10–30 m) around the reef during daylight hours.

### 2.4 Habitat classification and video interrogation

Habitats were initially classified from a still reference image taken from the beginning of each BRUVS deployment video, as per Speed et al. (2018). Visual estimates of coral cover (0%–100% rounded to the nearest 5%), complexity (low, medium, and high), and habitat type (sand, reef, or other) were estimated by eye, similar to other studies, (Espinoza et al., 2014; Malcolm, Jordan, & Smith, 2011; Speed et al., 2018; Tickler, Letessier, Koldewey, & Meeuwig, 2017) and replicated three times by independent recorders. Coral cover was then averaged across the three estimates to create an average percentage cover for each image. Discrepancies in either complexity or habitat type were decided using the most common category scored.

Video imagery was analyzed using the software EventMeasure (SeaGIS Pty. Ltd.), a purpose‐built event logger that allows an operator to record the number of fish observed and their species identification. To quantify the abundance of sharks and mesopredatory reef fishes, we recorded the maximum number of individuals of each species occurring in a single video frame (MaxN) during the entire video (Ellis & DeMartini, 1995; Meekan et al., 2006; Willis & Babcock, 2000). Each video was analyzed from the time the BRUVS landed on the seabed until sixty minutes of bottom time had occurred. This provided a standardized soak time for analyses.

### 2.5 Data processing

**Figure 2:**
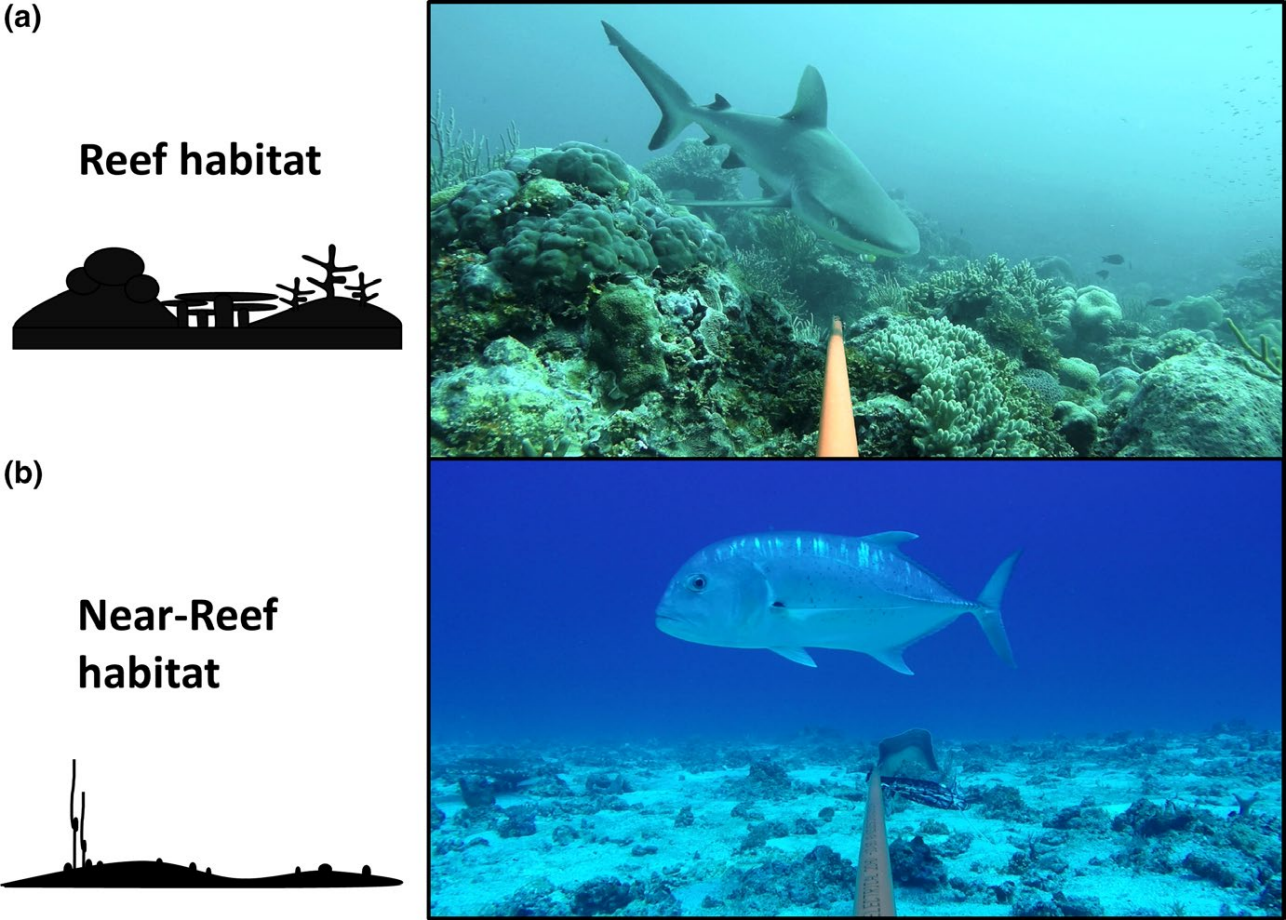
Images from footage of baited remote underwater video stations used to collect abundance data on reef predators in (a) reef habitats and (b) near‐reef habitats. Reef habitat was predominantly covered by hard and soft corals, wheras near‐reef was adjacent habitat that included sand, rubble, or algae cover. Species shown are (a) *Carcharhinus amblyrhynchos* and (b) *Caranx ignobilis*

As the composition and abundance of reef fish communities change with depth (e.g., Asher, Williams, & Harvey, 2017; Brokovich, Einbinder, Shashar, Kiflawi, & Kark, 2008; Fitzpatrick et al., 2012), we restricted our analyses to deployments on shallow reef habitats within a depth range of 10–30 m, although some shallower (<10 m) and deeper (>30 m) deployments were originally completed as part of a parallel study on elasmobranchs (Speed et al., 2018). Cross‐habitat comparisons of reef predator communities in 2004 and 2016 at Ashmore Reef included both reef habitat (hard and soft corals) and near‐reef habitat (sand, rubble, and consolidated limestone pavement) (Figure 2).

Common mesopredatory fishes found on the North West Shelf were included in the analysis of video data (Appendix S1). These were dominated by members of the families Lutjanidae, Lethrinidae, Serranidae, and Carangidae. Species that were difficult to identify were pooled into a single group with other, similar species of the same family. These included *Macolor niger* and *Macolor macularis* (*Macolor* spp), *Lethrinus olivaceus* and *Lethrinus* *microdon* (*Lethrinus* oli_micro), and *Plectropomus laevis* and *Plectropomus maculatus* (*Plectropomus* spp). All mesopredatory fishes were grouped into three size classes based on maximum obtainable lengths (TL) from regional estimates at Fishes of Australia http://fishesofaustralia.net.au/ (Bray & Gomon, 2018) and FishBase http://www.fishbase.org (Froese & Pauly, 2011), where regional TL estimates were unavailable (~22% of species). Mesopredatory fishes were classified as “small” (≤50 cm TL), “medium” (50–100 cm TL), and “large” (>100 cm TL) (e.g., Roff et al., 2019). Many sharks and large predatory teleosts are limited by gape width to consuming prey that are ~≤40% of their body length (Barley et al., 2017b; Bethea, Buckel, & Carlson, 2004), and up to as much as half their body length for some piscivores (Mihalitsis & Bellwood, 2017; Scharf, Juanes, & Rountree, 2000). As all species of shark recorded in our study attained at least 150 cm TL, the “small” size class of reef fishes was within the size range of prey for all species of shark, and the “medium” and “large” size classes were within the prey size range of apex species (>300 cm TL) such as tiger (*Galeocerdo cuvier*), bull (*Carcharhinus leucas*), and greater hammerhead (*Sphyrna mokarran*) sharks, although these larger species are not necessarily limited by gape width due to a variety of prey manipulation strategies (Braccini, 2008; Lucifora, García, Menni, & Escalante, 2006). The “large” size class of mesopredatory fishes (>100 cm TL) may occupy a size refuge from predators (at least at adult sizes) and will likely compete with sharks for prey (Roff et al., 2016a). Despite the occurrence of apex species at Ashmore Reef (Speed et al., 2018), these typically occurred in off‐reef (>1.5 km from reef edge) locations in deep water (>30 m). The focal group of sharks for our current study were considered to be reef residents (Heupel, Papastamatiou, Espinoza, Green, & Simpfendorfer, 2019) and were species that are site attached and can be found on reefs all year round. These included *Carcharhinus amblyrhynchos*, *Triaenodon obesus*, *Carcharhinus melanopterus*, *Stegostoma fasciatum*, and *Nebrius ferrugineus* (Appendix S2). All data were standardized to produce a value of MaxN per hour.

### 2.6 Data analyses

The first analysis focused on assessing whether there were differences in the community of predatory fishes between years in each of the two habitats (reef and near‐reef) at Ashmore Reef. Generalized linear models (GLMs) with negative binomial error structures were used to model abundance with factors that included year and a size class grouping (“small,” “medium,” “large,” and “sharks”), and their interaction. The variables' complexity and coral were found to be collinear in near‐reef data (Pearson Correlation = 0.6) and therefore not included in the same models. Changes in the mesopredatory fish communities were also assessed for Ashmore Reef between habitats and years using a principal component analysis (PCA) on scaled MaxN data for species that occurred on ≥10% of deployments. A permutational multivariate analysis of variance (PERMANOVA) test was then used to examine whether there were significant differences in composition of fish communities between years (2004 and 2016), habitats (reef and near‐reef), or between year and habitat combinations (interaction) at Ashmore Reef. Values of MaxN for each species were square root transformed prior to analyses to reduce the influence of dominant species, while still retaining the major differences in community structure according to abundances.

The second set of analyses investigated differences in the communities of mesopredatory fishes and reef sharks in reef habitats among reefs across the northwest region, and tested for an effect of protection status (fished vs. no‐take). Negative binomial GLMs were used to test for an effect of depth (10–30 m), coral cover (0%–100%), habitat complexity (low, medium, and high), and year (2004 & 2016), on the combined abundance of sharks and mesopredatory fish of all size classes (“small,” “medium,” “large”) across the northwest region of Western Australia. As data exploration within the region‐wide analyses revealed significant correlations between year with site and complexity with site (Pearson correlation = 0.8 and 0.5, respectively), these factors were not included within the same models for this analysis. A PERMANOVA test was used to examine whether there were significant differences in composition of fish communities between fished (Ashmore 2004 and Scott Reef 2016) and protected reefs (Ashmore 2016 and Rowley Shoals 2016) across the northwest region. Values of MaxN for each species were square root transformed prior to analyses.

During the data exploration stage for both sets of analyses, model residuals were plotted against fitted values and covariates to determine whether model assumptions were met (Zuur, Ieno, & Smith, 2007). Models for both sets of analyses were ranked using Akaike's information criterion corrected for small sample sizes (AIC_c_) and AIC_c_ weights (_w_AIC_c_) (Burnham & Anderson, 2002). Overdispersion was assessed using Pearson residuals, where a score of close to one was indicative of a lack of overdispersion (Zuur, Ieno, Walker, Saveliev, & Smith, 2009). Spatial autocorrelation was also assessed for the data from Ashmore Reef due to variation in locations of BRUVS deployments between years. This was done by plotting residuals from the top‐ranked model for both reef and near‐reef habitats against latitude and longitude (Figure S1) (Zuur et al., 2009).

Program R was used for all analyses (R Core Team, 2017) with packages *MASS* to fit negative binomial models (Venables & Ripley, 2002), *MuMIn* to rank models (Bartoń, 2013), *Visreg* to assess partial residuals (Breheny & Burchett, 2013), and *adonis* in the package *vegan* to run PERMANOVA tests (Anderson, 2001; McArdle & Anderson, 2001).

## 3 RESULTS

### 3.1 Comparison of communities of reef predators between reef and near‐reef habitats at Ashmore Reef

**Figure 3:**
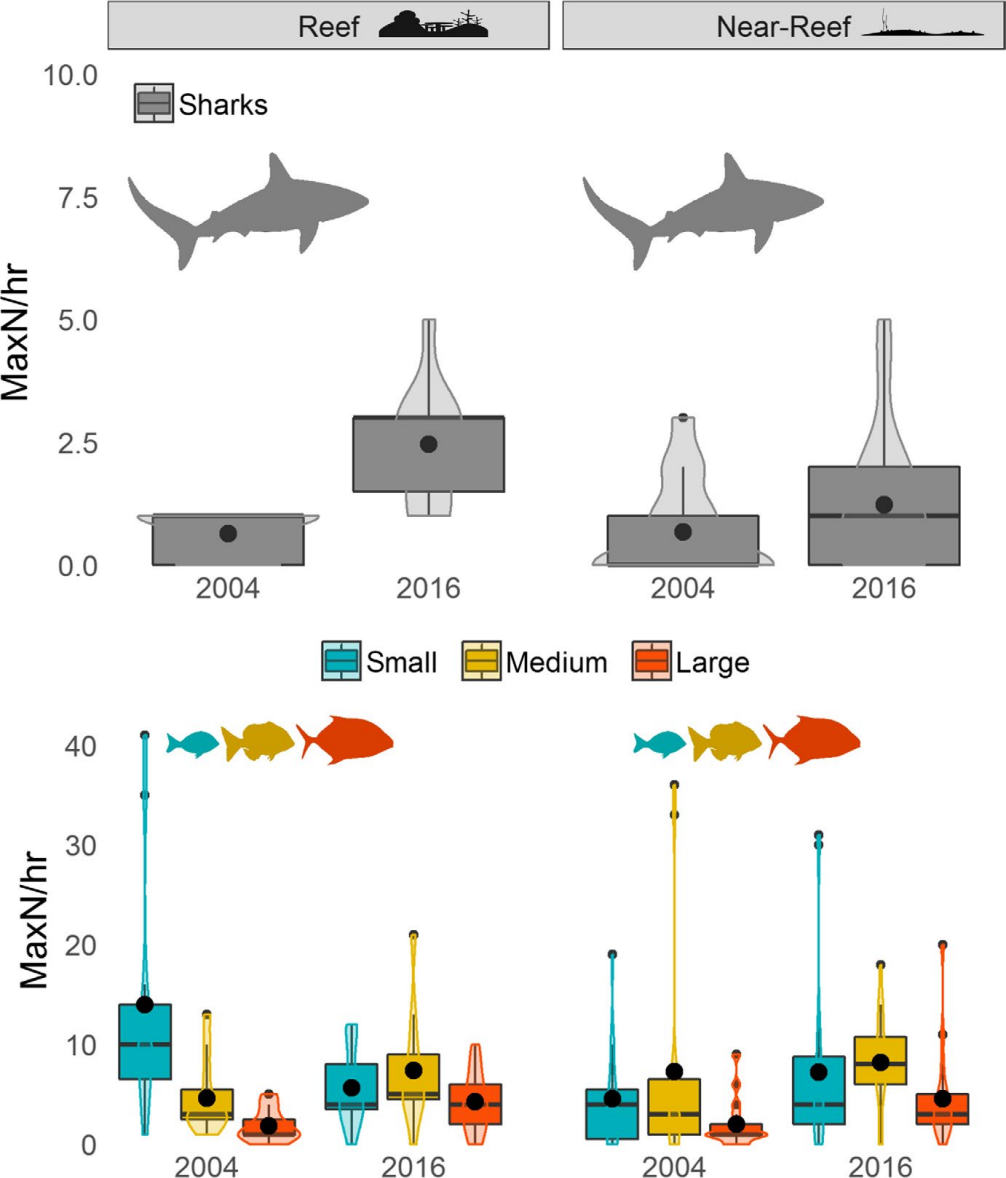
Abundance per hour for mesopredatory fishes and reef sharks at Ashmore Reef in reef and near‐reef habitats in 2004 and 2016. Boxplot centerline represents the median values, and top and bottom of box represent 25th and 75th percentiles, respectively. The black dot in the center of the box and violin plots represent the mean value per hour. Fish images for small, medium, and large are representative of species in that category

The top‐ranked negative binomial GLMs used to model the difference in total combined abundance of predatory fishes and shark communities between years at Ashmore Reef included year, size class, and depth (reef habitat model only) (Appendix S3). Both top‐ranked models showed limited evidence of overdispersion (1.00 for reef and 1.159 for near‐reef habitats) and explained 56% of the overall deviation explained (DE) for reef habitats and 30% of the overall DE for near‐reef habitats. Partial residual plots of abundance per hour for each size class derived from the top‐ranked model indicated differences between years and habitats for some groups (Figure S2). There was a large increase in shark abundance in reef habitat from 0.64 hr^−1^ ± 0.15 *SE* in 2004 to 2.45 hr^−1^ ± 0.37 *SE* in 2016, whereas a smaller increase occurred in near‐reef habitats from 0.67 hr^−1^ ± 0.25 *SE* to 1.22 hr^−1^ ± 0.34 *SE* (Figure 3). The increase in shark numbers was accompanied by a large decrease in the abundance of small mesopredatory fishes in reef habitats from 14.00 hr^−1^ ± 3.79 *SE* to 5.6 hr^−1^ ± 1.20 *SE* and a concomitant increase in large mesopredators from 1.82 hr^−1^ ± 0.48 *SE* to 4.27 hr^−1^ ± 0.93 *SE*. There was a smaller increase in the abundance of medium‐sized mesopredatory fishes in the reef habitat between 2004 and 2016 (4.63 hr^−1^ ± 1.13 *SE* to 7.36 hr^1^ ± 1.72 *SE*). Near‐reef fish communities had fewer noticeable changes in mean abundance of size classes, with only large mesopredatory fishes showing a clear increase between years from 2.00 hr^−1^ ± 0.65 *SE* in 2004 to 4.56 hr^−1^ ± 1.11 *SE* in 2016.

**Figure 4:**
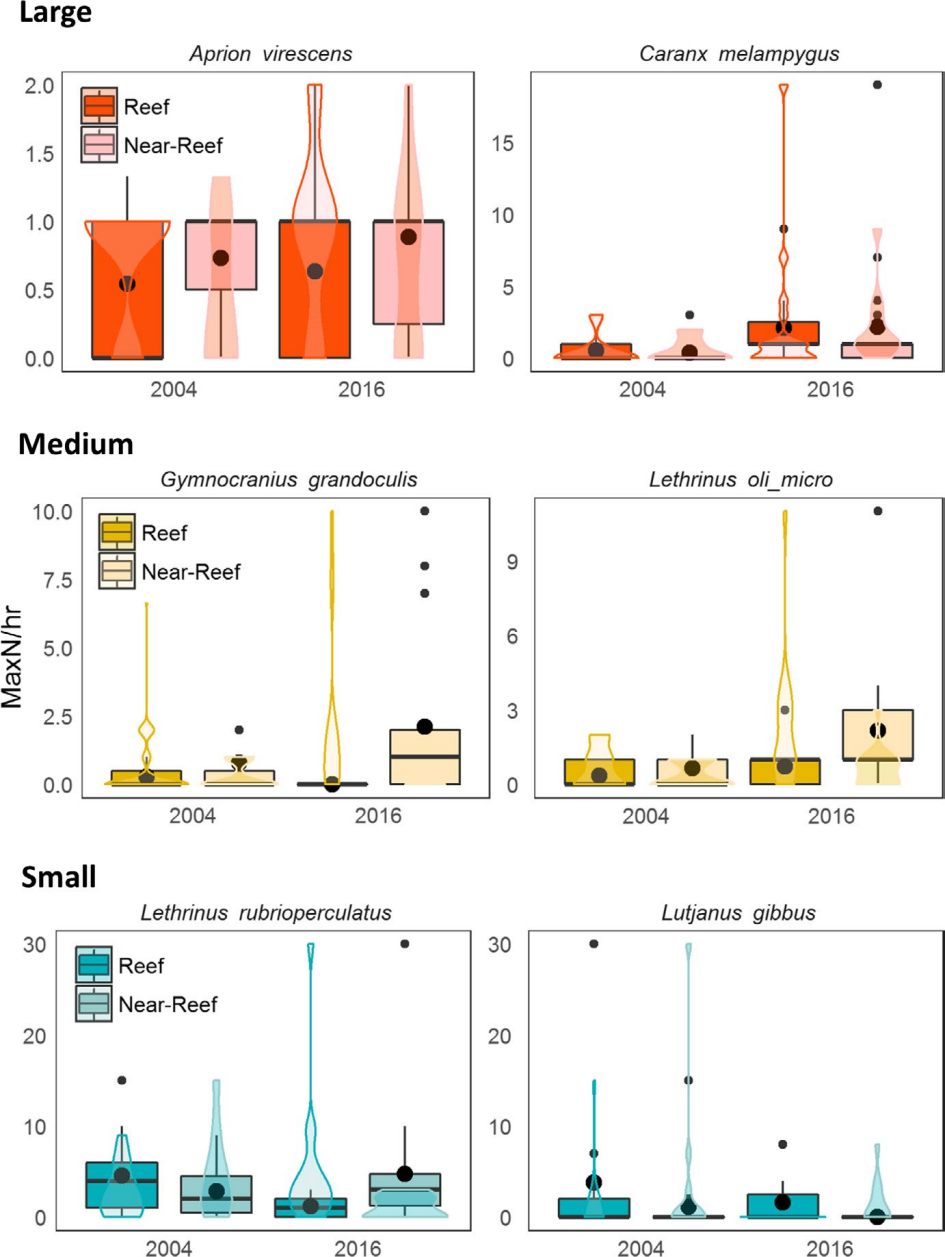
Abundance per hour of two of the most common and abundant species from each size class of mesopredatory reef fish between habitats and years at Ashmore Reef. Boxplot centerline represents the median values and top and bottom of box represent 25th and 75th percentiles, respectively. The black dot in the center of the box and violin plots represent the mean value per hour

Declines of small mesopredatory fishes in reef habitats between 2004 and 2016 were largely driven by changes in the abundance of lethrinids and lutjanids (e.g., *Lutjanus gibbus* and *Lethrinus rubrioperculatus*), whereas the increase in abundance of the large size class was primarily driven by representatives of the Carangidae (e.g., *Caranx melampygus*) (Figure 4). Similarly, the increase in abundance of large mesopredatory fishes in the near‐reef habitat was also driven by members of the Carangidae.

**Figure 5:**
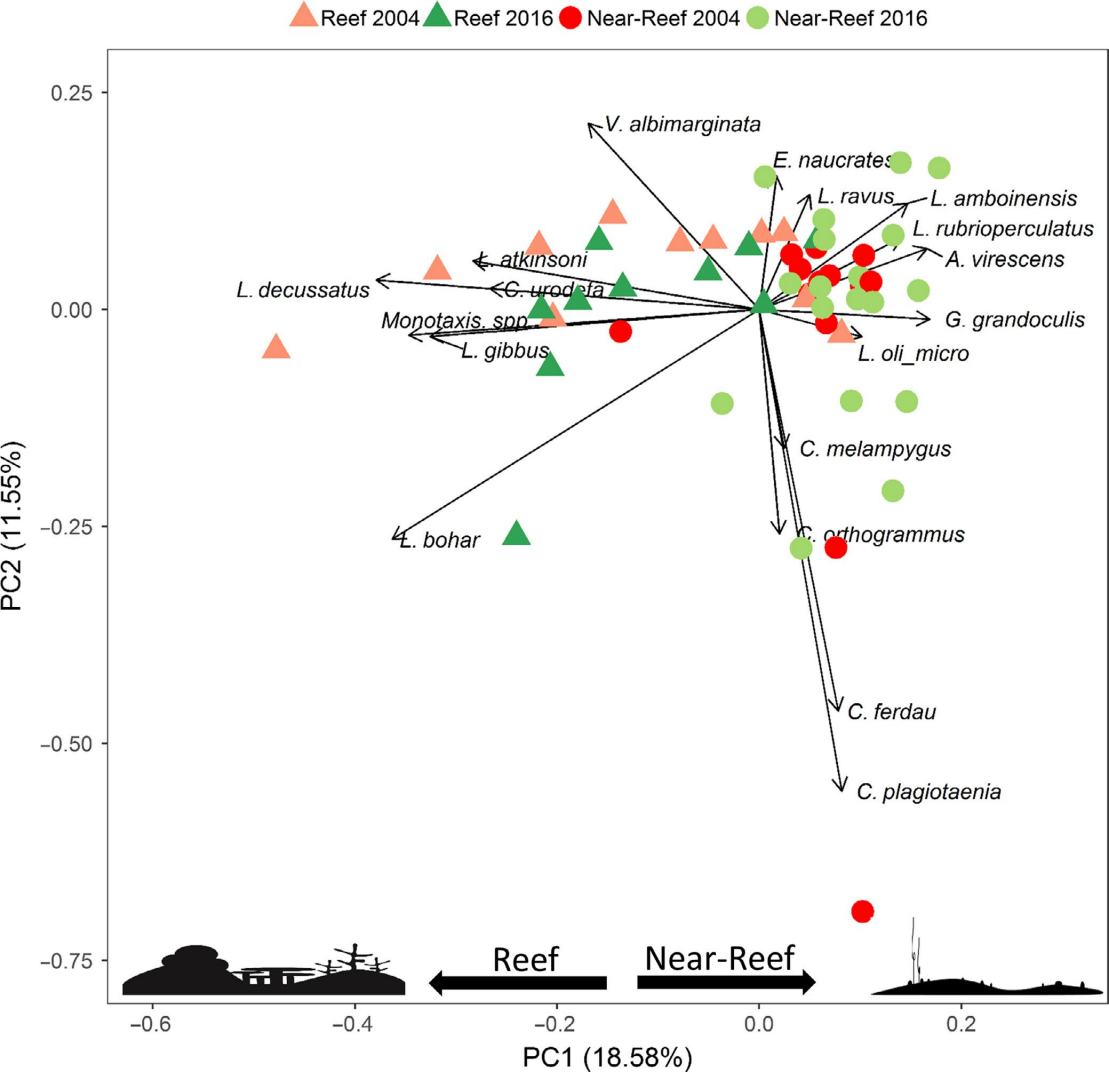
Principal component analysis of mesopredatory fish species occurring on ≥10% of BRUVS deployments at Ashmore Reef. Color scheme represents when Ashmore Reef was being illegally fished in 2004 in red and fully protected in 2016 in green

There was a significant difference in the structure and abundance of the mesopredatory fish community between habitats (*F* = 12.194, *p* < .001, perms = 999) and years (*F* = 3.057, *p* = .007) at Ashmore Reef (Table 1 and Figure 5).

**Table 1:** PERMANOVA output of assessment of mesopredatory fish size classes (small, medium, and large) between habitats (reef and near‐reef) and years (2004 and 2016) at Ashmore Reef. Significant factors are highlighted in bold font. MaxN values were square root transformed for PERMANOVA, with 9,999 permutations

	*df*	SumsOfSqs	MeanSqs	*F*.Model	*R* ^2^	Pr(>*F*)
**Habitat**	**1**	**2.295**	**2.295**	**12.194**	**0.184**	**<0.001**
**Year**	**1**	**0.576**	**0.575**	**3.057**	**0.046**	**0.007**
Habitat × Year	1	0.201	0.201	1.067	0.016	0.385
Residuals	50	9.411	0.188	0.754		

### 3.2 Regional comparison of communities of mesopredatory fishes in reef habitats

**Figure 6:**
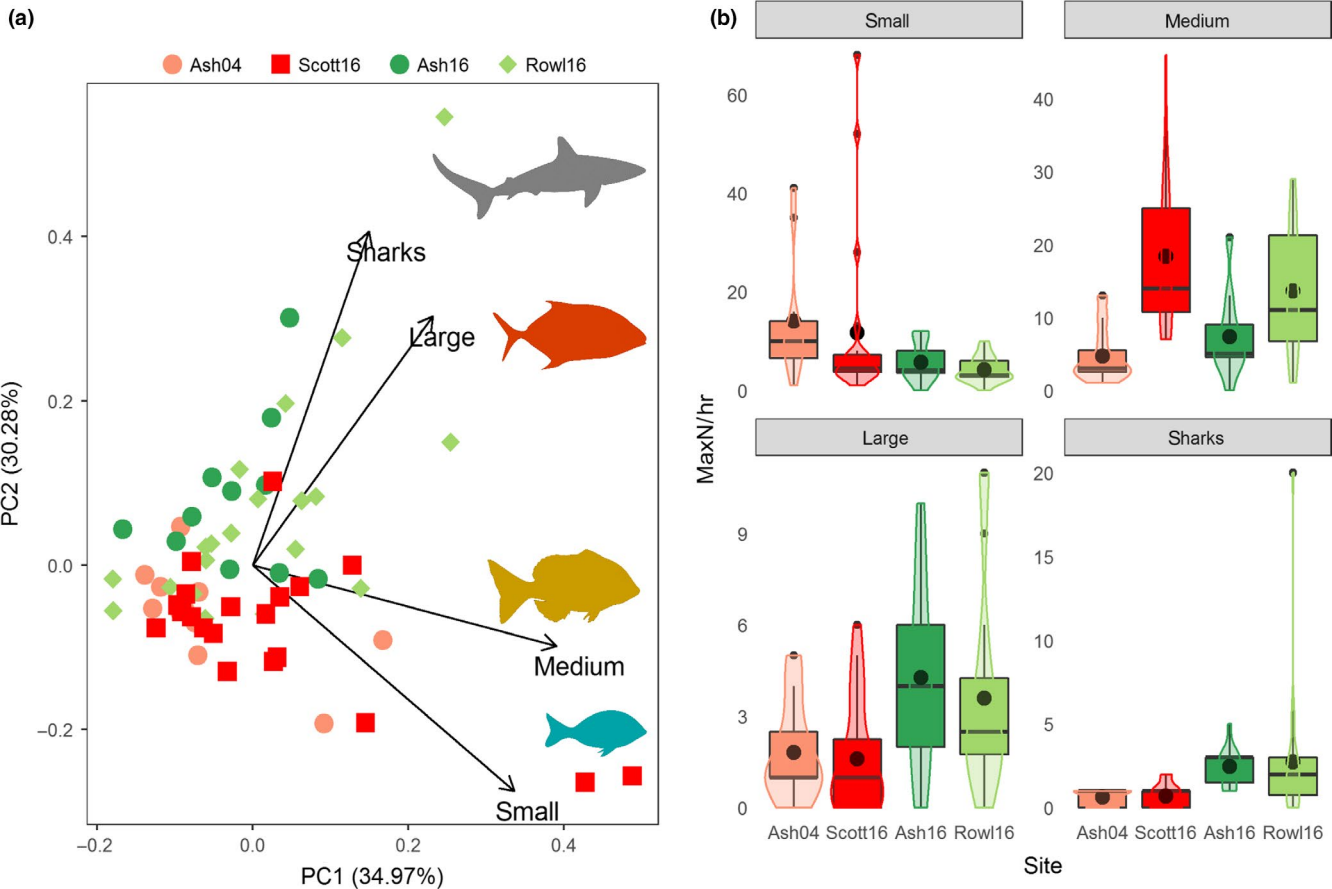
(a) Principal component analysis of size classes of mesopredatory fishes and sharks in reef slope habitat between protected versus fished reefs in northwestern Australia, and (b) abundance of mesopredatory fishes (small, medium, and large size classes) and sharks (all species combined) observed per hour of baited video deployments on the reef slope habitat at study sites. Boxplot centerline represents the median values, and top and bottom of box represent 25th and 75th percentiles, respectively. The black dot in the center of the box and violin plots show the mean value per hour. Size classes of mesopredatory fish were small (≤50 cm TL), medium (50–100 cm TL), and large (>100 cm TL). Ashmore Reef 2004 (Ash04) and Scott Reefs 2016 (Scott16) are colored shades of red to represent the treatment “fished”, wheras Ashmore Reef 2016 (Ash16) and the Rowley Shoals 2016 (Rowl16) are colored shades of green to represent the treatment “protected”

The top‐ranked negative binomial GLM used to model differences in predatory fish communities among reefs in the region showed slight evidence of overdispersion (1.26). The abundance of communities of predatory fishes (sharks and mesopredatory fishes) was strongly associated with the variable site, as indicated by the top‐ranked negative binomial GLM (wAICc = 0.38, D.E. = 54.48), although depth and coral cover were also important explanatory variables with similar ranked models falling within two AIC_c_ (Table 2 and Figure S3). Partial residual plots of abundance per hour for each size class derived from the top‐ranked model indicated differences among reefs for some groups (Figure S4). Principal component analysis showed a clear separation in predator communities between fished (Ashmore Reef 2004 and Scott Reef 2016) and protected reefs (Ashmore 2016 and Rowley Shoals 2016) (Figure 6a). The effect of protection status on community composition was significant (*F* = 6.19, *p* < .001, perms = 9,999). Separation was driven by higher abundances of sharks and large mesopredatory fishes at protected reefs, whereas smaller mesopredatory fishes were more abundant at fished reefs (Figure 6a,b).

**Table 2:** Model outputs for negative binomial generalized linear models of combined MaxN of all predatory fishes and sharks in reef habitats of northwestern Australia. The explanatory variable “Site” includes Ashmore Reef in 2004 and 2016, and Scott Reefs and Rowley Shoals in 2016. “Group” was a factorial variable that included levels for fish size categories: small, medium, large, and sharks. The top‐ranked model is highlighted in bold font

Model	*K*	logLik	AICc	ΔAICc	ΔAICc	D.E. (%)	Null dev.	Resid. Dev.
**Site × Group**	**17**	**−621.83**	**1,280.31**	**0.00**	**0.38**	**54.48**	**565.26**	**257.31**
Site × Group + Depth + Coral	19	−619.51	1,280.35	0.03	0.37	55.40	579.48	258.45
Site × Group + Depth	18	−621.06	1,281.11	0.80	0.25	54.79	570.69	258.01
Depth × Site	9	−715.79	1,450.34	170.02	0.00	4.76	290.48	276.65
Coral × Site	9	−716.15	1,451.06	170.74	0.00	4.51	289.98	276.90
Coral	3	−719.85	1,445.79	165.48	0.00	1.92	282.69	277.27
Depth	3	−719.87	1,445.83	165.52	0.00	1.90	282.65	277.28
Complexity	3	−720.34	1,446.78	166.46	0.00	1.57	281.83	277.41
Year	3	−721.92	1,449.94	169.63	0.00	0.43	278.82	277.61
Intercept	2	−722.53	1,449.10	168.79	0.00	0.00	277.69	277.69

*Lutjanus gibbus* was one of the small species of mesopredatory fishes that was more common at fished reefs (Ashmore Reef in 2004 and Scott Reef in 2016) and typically occurred in schools (Figure S5). In contrast, other small species such as *Lutjanus decussatus* remained in similar abundances across reefs in the northwest, as did some of the medium‐size class of fishes such as *Lutjanus bohar* (Figure S5). There were noticeable differences in abundances of large mesopredatory species across the reefs, with *C. melampygus* and *Plectropomus* spp. complex more common at nonfished reefs at Ashmore in 2016 and the Rowley Shoals in 2016 (Figure S5).

## 4 DISCUSSION

Changes in the abundance of small mesopredatory fishes following the recovery of reef sharks and large mesopredatory fishes on Ashmore Reef provide support for the role that these predators play in structuring fish assemblages and corroborate findings of earlier spatial comparisons in this region (See Barley et al., 2017b; Ruppert et al., 2013). At the reef scale, shark recovery in reef habitats at Ashmore Reef was accompanied by a 2.5‐fold reduction in the abundance of small mesopredatory fishes (<50 cm TL), a result that is consistent with other studies that have observed up to fourfold negative reductions in mesopredators with increasing numbers of apex predators (Ritchie & Johnson, 2009). Conversely, increases of similar magnitudes in the abundance of mesopredators have been observed in other communities of coral reef fishes where larger predators have been reduced in abundance through fishing (Graham, Evans, & Russ, 2003; Dulvy, Polunin, Mill, & Graham, 2004; Myers, Baum, Shepard, Powers, & Peterson, 2007, although see; Grubbs et al., 2016).

The shift in fish community structure at Ashmore Reef has now resulted in abundances and size structures of mesopredatory fishes that are comparable to the Rowley Shoals, a locality that has not been subjected to fishing for almost three decades and provides a baseline for a “pristine” reef in this region of the Indian Ocean. The high abundance and biomass of sharks and large mesopredatory fishes found at the Rowley Shoals are typical of coral reefs in other regions that are also subject to little fishing pressure (e.g., DeMartini, Friedlander, Sandin, & Sala, 2008; Rizzari et al., 2015; Sandin et al., 2008; Ward‐Paige, Flemming, & Lotze, 2010; Williams et al., 2010).

The decline in the abundance of small mesopredatory fishes with the recovery of shark populations at Ashmore Reef was also accompanied by an increase in the abundance of larger mesopredatory fishes. It is possible that this increase in larger species also contributed to the decline in smaller mesopredators, either through competition or direct predation, although disentangling these effects was not possible in our study. However, in near‐reef habitats, there was a much smaller increase in shark numbers and a considerable rise in the numbers of large mesopredators, similar to that occurring on the reef. In this habitat, numbers of small mesopredators only increased slightly through time, suggesting that changes in the abundance of large mesopredators had a weaker impact on the abundance of small mesopredators than sharks. It is likely that many of the increases in abundances of larger mesopredatory fishes and reef sharks we observed at Ashmore Reef were as a result of a reduction in illegal fishing pressure through increased enforcement of the no‐take reserve (Speed et al., 2018).

Changes in the abundance of small mesopredators were limited to mostly lutjanids and lethrinids, which largely overlapped with those species identified by Ruppert et al. (2013) and Barley et al. (2017b) as likely to be subjected to mesopredator release. The concept of sharks influencing the abundance of these mesopredators by direct predation has been questioned because they are rarely found in gut contents of sharks (Frisch et al., 2016; Roff et al., 2016b), although this observation provides little basis on which to judge the diet and trophic role of sharks or their impacts on the abundance or behaviors of reef fishes (Hammerschlag, 2019). The stomach contents of sharks caught in many studies are often empty or contain fish that are too digested to be identified, so that even occasional findings of mesopredatory species (e.g., Brewer, Blaber, Salini, & Farmer, 1995; Randall, 1977; Stevens, 1984) are likely to be important indicators of diet.

There is, however, a possibility that the presence of predators might influence the abundance of prey on BRUVS. Some studies have found that the presence of larger predators in the field of view of BRUVS can negatively affect the overall relative abundance of prey over reduced sampling periods (30 min), although this effect appears to be species‐dependent (e.g., Dunlop, Marian Scott, Parsons, & Bailey, 2015; Klages, Broad, Kelaher, & Davis, 2014). In contrast, a recent study by Coghlan, McLean, Harvey, and Langlois (2017) found that the abundance of a predatory reef fish did not influence the abundance of other, smaller species over longer sampling periods (60 min). Conservative metrics such as MaxN (Colton & Swearer, 2010) may be relatively robust for smaller species when estimated over long soak times (60 min+) (Klages et al., 2014). Indeed, we found no support in our study for a negative effect of shark presence on the relative abundance of smaller mesopredatory fish over 60‐min sampling periods (Appendix S4).

In addition to a shift in abundance of size classes between years at Ashmore Reef, there was also evidence for redistribution of some species between reef and near‐reef habitats, possibly as a response to the increase in shark numbers. For example, in 2004 when sharks were in low numbers at Ashmore Reef, the small piscivore *L. rubrioperculatus* (Trianni & Tenorio, 2012) occurred more commonly in the reef habitat, whereas in 2016, once shark populations had recovered, this species was more abundant in near‐reef habitats (largely sand and rubble) where there had been a much smaller increase in shark numbers. Total abundances of this species differed little between 2004 and 2016, suggesting that redistribution may have contributed to these changes. If this is the case, then redistribution may represent a shift by prey to less profitable habitat as a means of predator avoidance, a behavioral pattern reported for prey species in other marine habitats (e.g., Frid, Dill, Thorne, & Blundell, 2007; Heithaus & Dill, 2002; Heithaus & Dill, 2006; Wirsing, Heithaus, & Dill, 2007).

Increases in large mesopredators were mostly driven by carangids, including species such as the bluefin trevally (*C. melampygus*), which are known to have a largely piscivorous diet (Dale, Meyer, & Clark, 2011), are highly mobile (Asher et al., 2017) and are likely to be susceptible to the fishing techniques in near‐reef environments that are used by Indonesian fishermen to target sharks (Russell & Vail, 1988). Such increases in the abundance or biomass of larger species of mesopredators that are targeted by fishing after the creation of no‐take reserve are a common pattern in coral reef environments (Edgar et al., 2014; Russ & Alcala, 1996). No clear spatio‐temporal trends in the abundance of medium‐sized mesopredatory fishes (50–100 cm TL) emerged from our study. This is perhaps not surprising, given that the endpoints of our size categories were based on likely prey sizes for reef sharks. Species at the smaller end of the medium‐size spectrum (e.g., 50–75 cm TL) may be prey to large‐bodied mesopredatory fishes and sharks, whereas species at the opposite end of the size spectrum (75–100 cm TL) could act as predators of small‐sized mesopredatory fishes and possibly as competitors of large‐bodied fishes and sharks (e.g., Frisch et al., 2014). As populations recovered and sharks attained adult sizes, the size spectra of fishes that constituted prey are likely to have also changed through time (Shin & Cury, 2004), although dedicated studies using stereo‐BRUVS would be required to confirm this.

The similarities between abundances of reef sharks and the structure of mesopredatory reef fish communities now present at Ashmore Reef and the protected Rowley Shoals implies that both the reef shark and mesopredatory fish communities have largely recovered from illegal fishing in the eight years since enforcement of the no‐take status of the reef. However, it must be acknowledged that our study represents two snapshots in time and therefore requires further sampling to confirm that long‐term patterns in recovery continue. Nevertheless, the rapid rate of recovery in shark and mesopredatory fish numbers at Ashmore Reef will be encouraging for managers seeking time frames in which to predict for stakeholders when impacts from conservation actions might occur.

## CONFLICT OF INTEREST

None declared.

## AUTHOR CONTRIBUTION

MGM, CWS, and MR conceived and designed the project. MGM, CWS, and KC collected the data. BV, KC, MR, and CWS performed BRUVS footage processing. CWS statistically analyzed the data. MGM, CWS, MR, KC, and BV wrote and edited the manuscript.

## Data Availability

All data used in this study are available at Global Archive: https://globalarchive.org/geodata/data/project/get/234.
