# Supplementary material for: Protection from illegal fishing and shark recovery restructures mesopredatory fish communities on a coral reef
Source: Ecol Evol. 2019 Aug 20;9(18):10553–66. doi: 10.1002/ece3.5575 (PMC6787830; doi:10.1002/ece3.5575)
Supplement: Supplementary file 1 [file ECE3-9-10553-s001.docx]

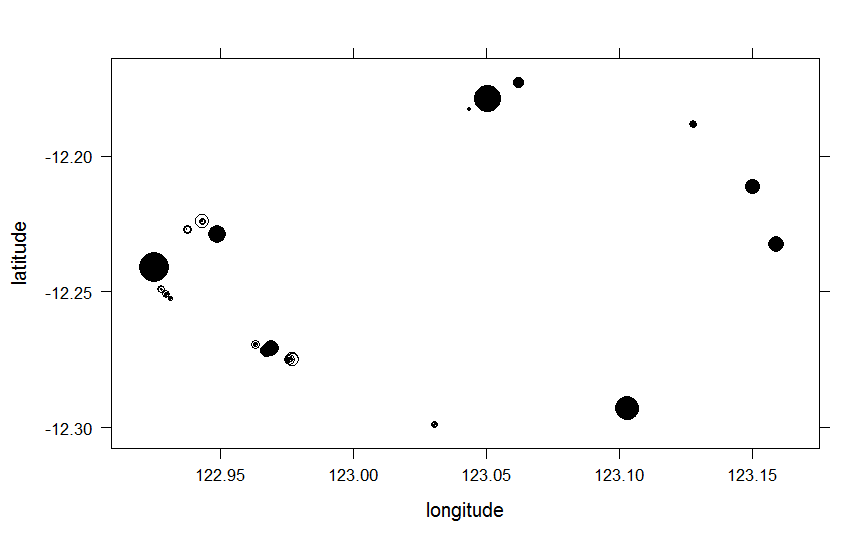

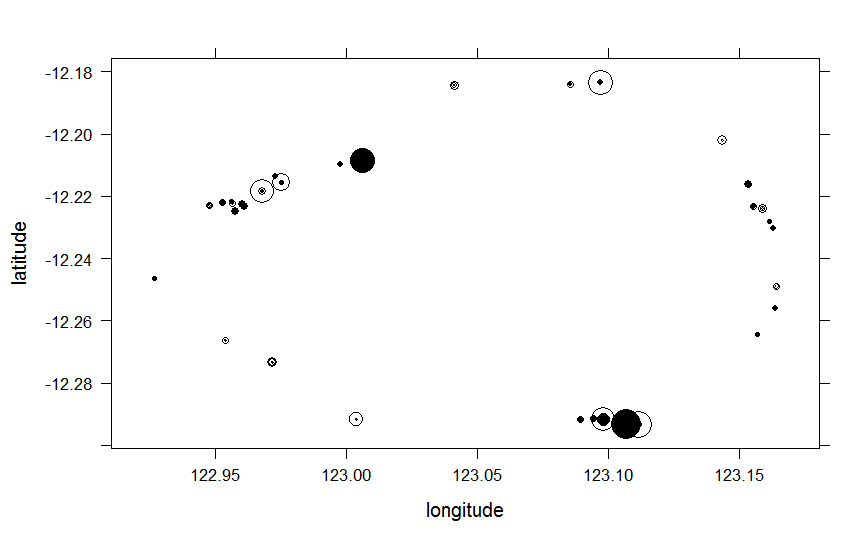


**A**

**B**

**Figure S1.** Residuals of the top-ranked negative binomial GLM plotted in space (latitude & longitude) for A) reef habitat samples and B) near-reef habitat samples. The larger the dot, the larger the residual. Solid dots are negative values and open are positive.
