# Supplementary material for: Protection from illegal fishing and shark recovery restructures mesopredatory fish communities on a coral reef
Source: Ecol Evol. 2019 Aug 20;9(18):10553–66. doi: 10.1002/ece3.5575 (PMC6787830; doi:10.1002/ece3.5575)
Supplement: Supplementary file 2 [file ECE3-9-10553-s002.docx]

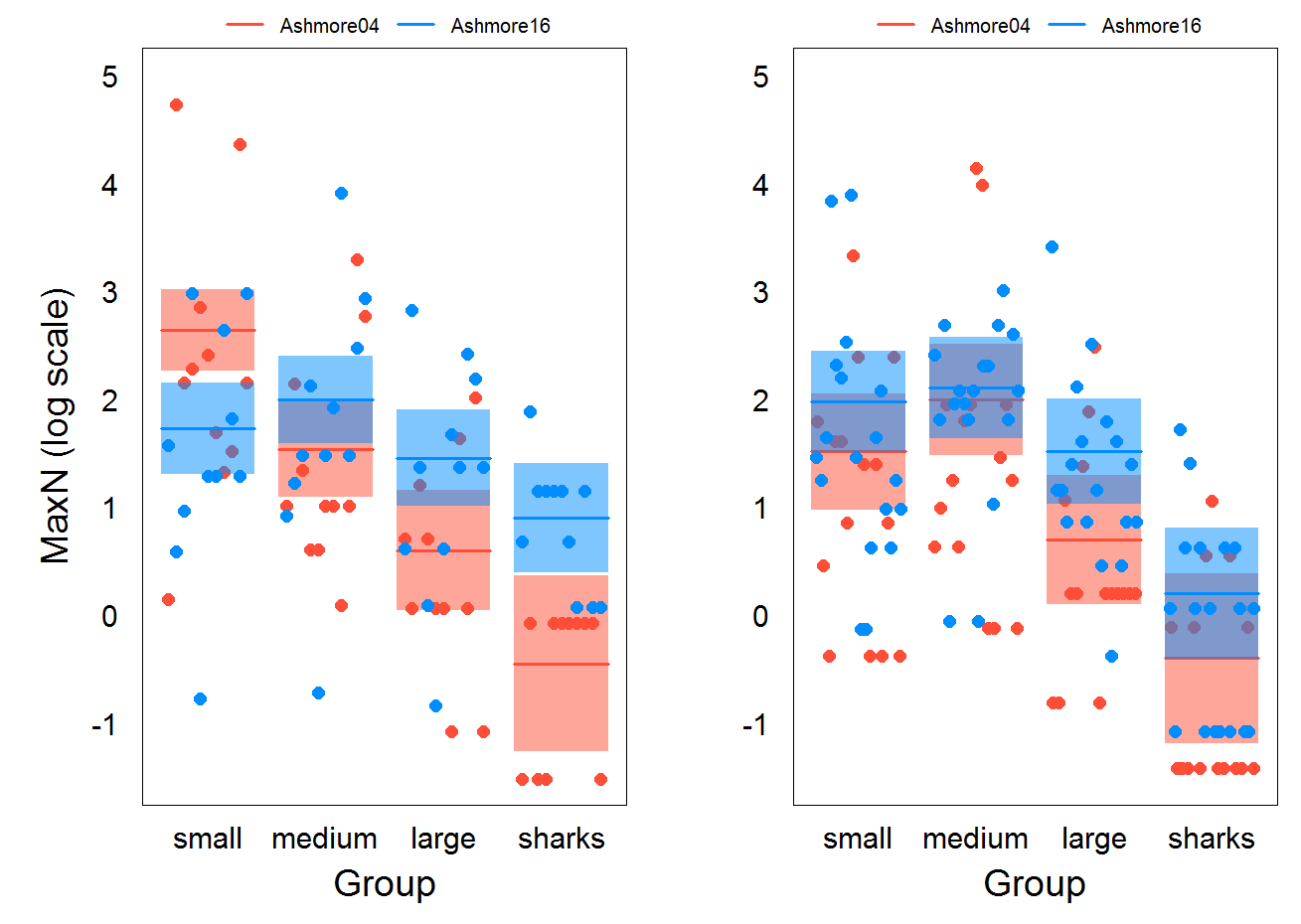


**A**

**B**

**Figure S2.** Partial residual plots of MaxN values per hour for mesopredatory fish and sharks derived from top-ranked Negative Binomial GLM including habitat and year at Ashmore Reef. Panel A represents reef habitat and panel B represents near-reef habitat.
