# Supplementary material for: Protection from illegal fishing and shark recovery restructures mesopredatory fish communities on a coral reef
Source: Ecol Evol. 2019 Aug 20;9(18):10553–66. doi: 10.1002/ece3.5575 (PMC6787830; doi:10.1002/ece3.5575)
Supplement: Supplementary file 3 [file ECE3-9-10553-s003.docx]

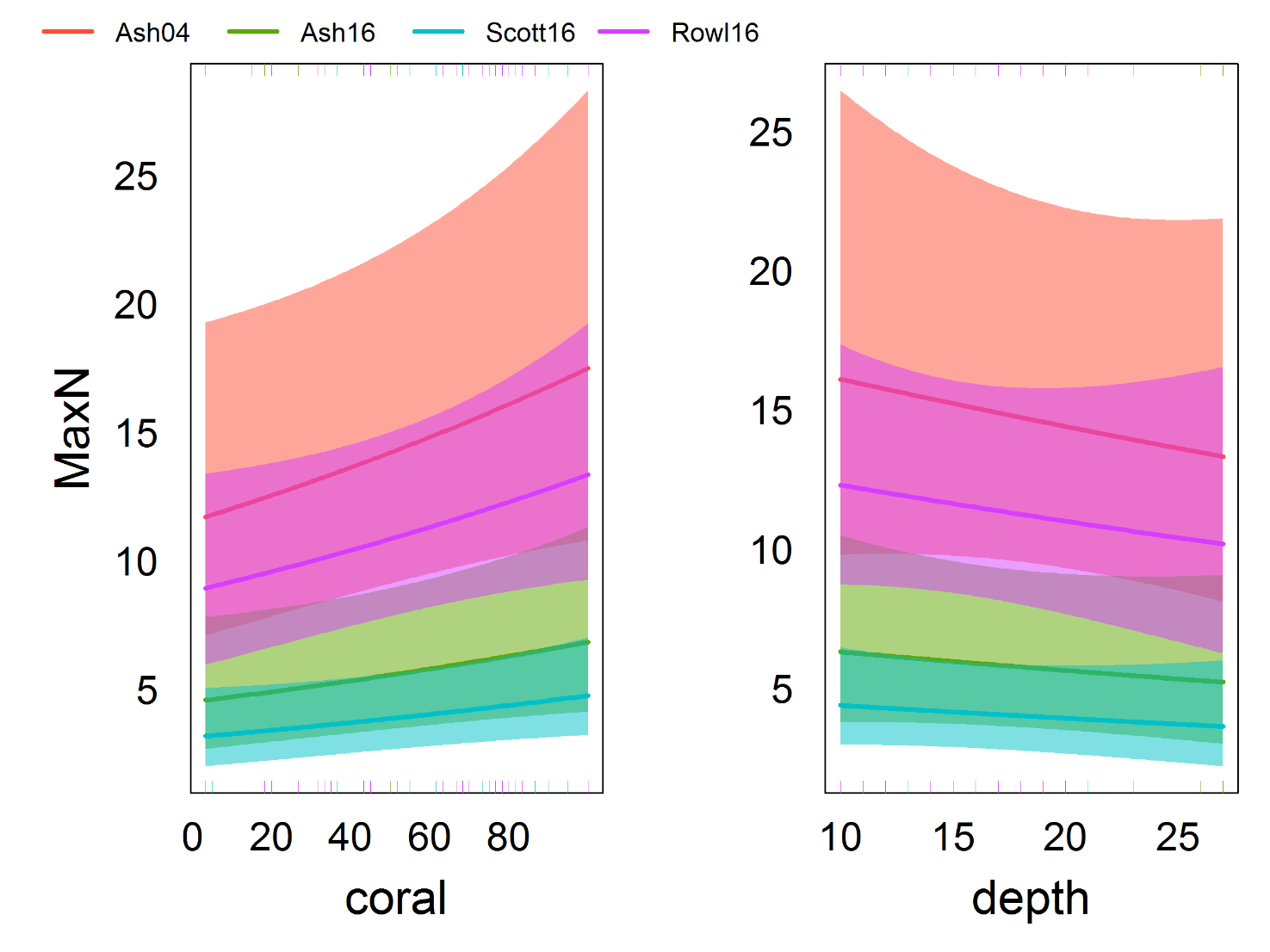


**Figure S3.** Partial residual plots of combined MaxN values for all mesopredatory fish and sharks from the full Negative Binomial GLM predictor variables coral cover and depth in reef habitats in north Western Australia.
