# Supplementary material for: Protection from illegal fishing and shark recovery restructures mesopredatory fish communities on a coral reef
Source: Ecol Evol. 2019 Aug 20;9(18):10553–66. doi: 10.1002/ece3.5575 (PMC6787830; doi:10.1002/ece3.5575)
Supplement: Supplementary file 4 [file ECE3-9-10553-s004.docx]

**
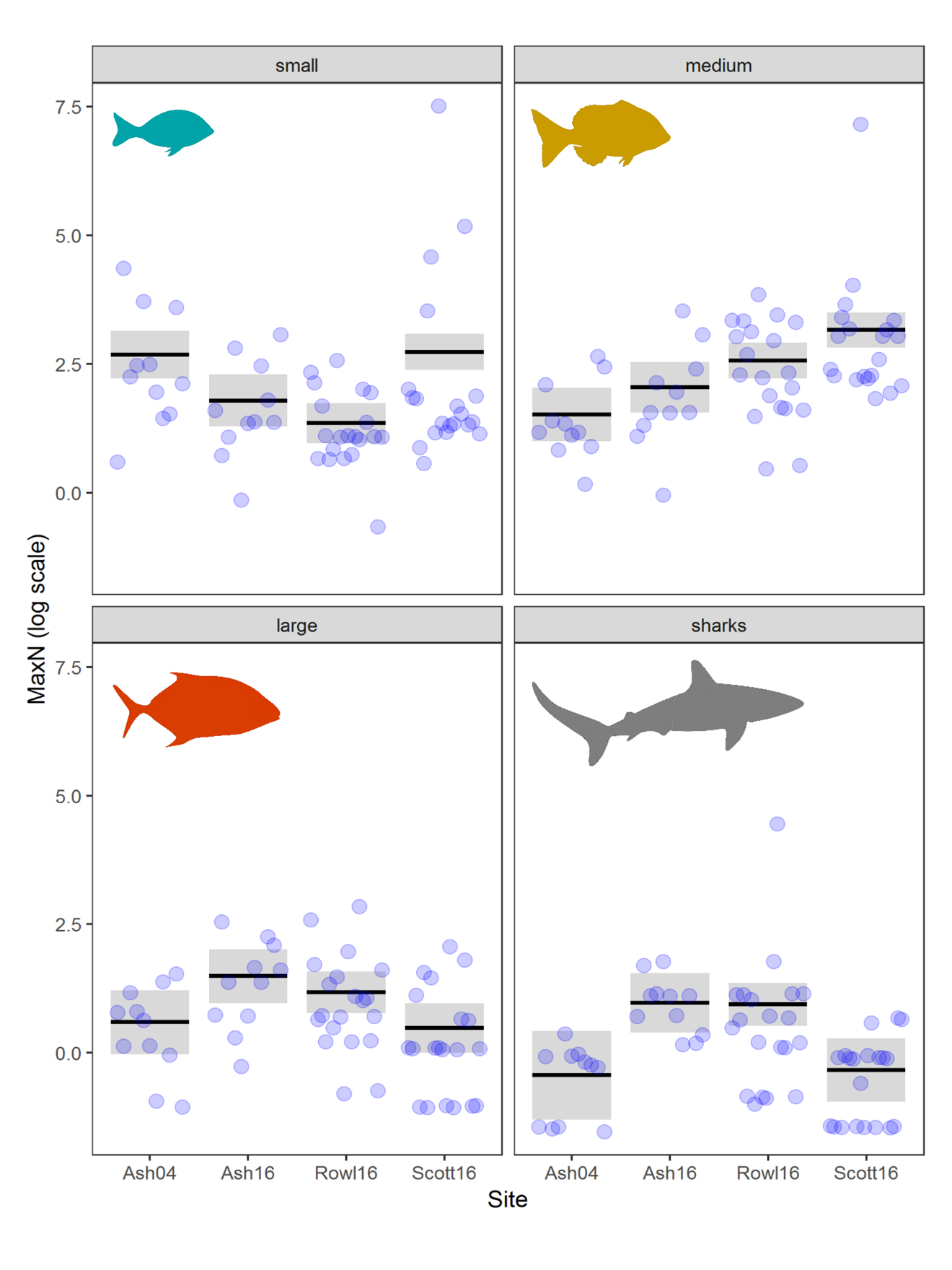
**

**Figure S4.** Partial residual plots of MaxN values per hour for mesopredators derived from top-ranked Negative Binomial GLM including site (Ashmore Reef 2004 and 2016, Rowley Shoals 2016, and Scott Reefs 2016, and 'Group’ (small, medium, large, and sharks).
