# Supplementary material for: Protection from illegal fishing and shark recovery restructures mesopredatory fish communities on a coral reef
Source: Ecol Evol. 2019 Aug 20;9(18):10553–66. doi: 10.1002/ece3.5575 (PMC6787830; doi:10.1002/ece3.5575)
Supplement: Supplementary file 5 [file ECE3-9-10553-s005.docx]

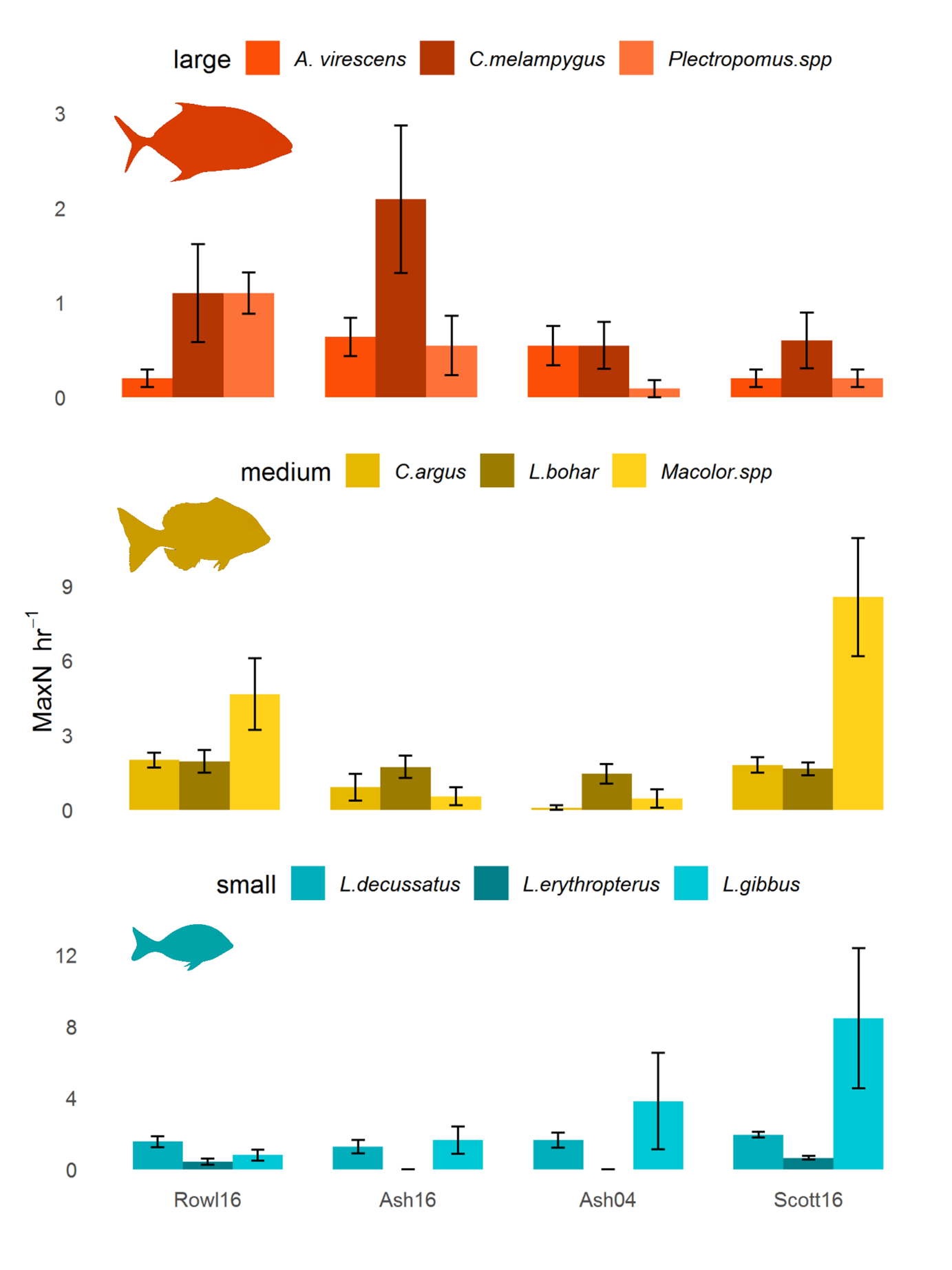


**Figure S5.** Average abundance (± SE) of three commonly occurring species from each size class of mesopredatory fish in reef habitats in offshore north Western Australia.
