# Supplementary material for: Protection from illegal fishing and shark recovery restructures mesopredatory fish communities on a coral reef
Source: Ecol Evol. 2019 Aug 20;9(18):10553–66. doi: 10.1002/ece3.5575 (PMC6787830; doi:10.1002/ece3.5575)
Supplement: Supplementary file 6 [file ECE3-9-10553-s006.docx]

APPENDICIES

**Appendix 1.** Common mesopredatory reef fish species list for north Western Australia. Species maximum lengths were obtained primarily from Fishes of Australia <http://fishesofaustralia.net.au/> and also Fishbase (<http://www.fishbase.org>). Size classes were assigned based on maximum size attainable.

| **Family** | **Species** | **Max. length** | **Size class** | **Source** |
| --- | --- | --- | --- | --- |
| Serranidae | *Aethaloperca rogaa* | 60 cm TL | medium | Fishes of Australia |
| Lutjanidae | *Aphareus furca* | 70 cm FL | medium | Fishes of Australia |
| Lutjanidae | *Aprion virescens* | 112 cm TL | large | Fishes of Australia |
| Carangidae | *Carangoides ferdau* | 70.0 cm TL | medium | FishBase |
| Carangidae | *Carangoides orthogrammus* | 75.0 cm TL | medium | Fishes of Australia |
| Carangidae | *Carangoides plagiotaenia* | 50.0 cm TL | small | FishBase |
| Carangidae | *Caranx ignobilis* | 180 cm TL | large | Fishes of Australia |
| Carangidae | *Caranx lugubris* | 100 cm TL | medium | Fishes of Australia |
| Carangidae | *Caranx melampygus* | 120 cm TL | large | Fishes of Australia |
| Serranidae | *Cephalopholis argus* | 60 cm TL | medium | Fishes of Australia |
| Serranidae | *Cephalopholis urodeta* | 28 cm TL | small | Fishes of Australia |
| Echeneidae | *Echeneis naucrates* | 110 cm TL | large | Fishes of Australia |
| Serranidae | *Epinephelus areolatus* | 50 cm TL | small | Fishes of Australia |
| Serranidae | *Epinephelus chlorostigma* | 80 cm TL | medium | Fishes of Australia |
| Serranidae | *Epinephelus fasciatus* | 50 cm TL | small | Fishes of Australia |
| Serranidae | *Epinephelus fuscoguttatus* | 120 cm TL | large | Fishes of Australia |
| Serranidae | *Epinephelus maculatus* | 60.5 cm TL | medium | FishBase |
| Serranidae | *Epinephelus merra* | 35 cm TL | small | Fishes of Australia |
| Serranidae | *Epinephelus polyphekadion* | 90 cm SL | medium | Fishes of Australia |
| Carangidae | *Gnathanodon speciosus* | 120 cm TL | large | Fishes of Australia |
| Lethrinidae | *Gymnocranius grandoculis* | 80.0 cm TL | medium | FishBase |
| Scombridae | *Gymnosarda unicolor* | 248 cm FL | large | Fishes of Australia |
| Labridae | *Halichoeres hortulanus* | 27.0 cm TL | small | FishBase |
| Lethrinidae | *Lethrinus amboinensis* | 70 cm TL | medium | Fishes of Australia |
| Lethrinidae | *Lethrinus atkinsoni* | 50.0 cm TL | small | FishBase |
| Lethrinidae | *Lethrinus erythracanthus* | 70 cm TL | medium | Fishes of Australia |
| Lethrinidae | *Lethrinus erythropterus* | 50 cm TL | small | Fishes of Australia |
| Lethrinidae | *Lethrinus nebulosus* | 94 cm TL | medium | Fishes of Australia |
| Lethrinidae | *Lethrinus obsoletus* | 60 cm TL | medium | Fishes of Australia |
| Lethrinidae | *Lethrinus oli_micro** | 100 cm TL | medium | Fishes of Australia |
| Lethrinidae | *Lethrinus ravus* | 25.0 cm SL | small | FishBase |
| Lethrinidae | *Lethrinus rubrioperculatus* | 50.0 cm TL | small | FishBase |
| Lethrinidae | *Lethrinus semicinctus* | 35.0 cm TL | small | FishBase |
| Lethrinidae | *Lethrinus xanthochilus* | 70 cm FL | medium | Fishes of Australia |
| Lutjanidae | *Lutjanus bohar* | 90 cm TL | medium | Fishes of Australia |
| Lutjanidae | *Lutjanus decussatus* | 30 cm TL | small | Fishes of Australia |
| Lutjanidae | *Lutjanus gibbus* | 50 cm TL | small | Fishes of Australia |
| Lutjanidae | *Lutjanus kasmira* | 40 cm TL | small | Fishes of Australia |
| Lutjanidae | *Lutjanus monostigma* | 60 cm TL | medium | Fishes of Australia |
| Lutjanidae | *Lutjanus vitta* | 40 cm TL | small | Fishes of Australia |
| Lutjanidae | *Macolor spp** | 75.0 cm TL | medium | FishBase |
| Lethrinidae | *Monotaxis spp** | 60 cm TL | medium | Fishes of Australia |
| Cirrhitidae | *Paracirrhites forsteri* | 22.0 cm TL | small | FishBase |
| Serranidae | *Plectropomus spp** | 125 cm TL | large | Fishes of Australia |
| Rachycentridae | *Rachycentron canadum* | 200 cm FL | large | Fishes of Australia |
| Scombridae | *Scomberomorus commerson* | 240 cm FL | large | Fishes of Australia |
| Scombridae | *Scomberomorus queenslandicus* | 100 cm FL | medium | Fishes of Australia |
| Sphyraenidae | *Sphyraena barracuda* | 200 cm TL | large | Fishes of Australia |
| Serranidae | *Variola albimarginata* | 65 cm TL | medium | Fishes of Australia |

*****These are species complexes where multiple species were grouped due to difficulties with consistent identification from BRUVS. These species complexes include: Lethrinus oli_micro (*olivaceous* & *microdon*), Monotaxis spp. (*grandoculis* & *heterodon*), Macolor spp (*macularis* & *niger*), and *Plectropomus spp*. (*laevis* & *maculatus*).

**Appendix 2.** Percentage contribution to abundance of reef shark species observed during baited remote underwater video station surveys in north Western Australia.

| **Reef** | **Habitat** | **Species** | **Year** | |
| --- | --- | --- | --- | --- |
|  |  |  | **2004** | **2016** |
| **Ashmore Reef** | Near-reef | *Carcharhinus amblyrhynchos* | 30.0 | 81.8 |
|  |  | *Triaenodon obesus* | 70.0 | 18.2 |
|  | Reef | *Carcharhinus amblyrhynchos* | 14.3 | 66.7 |
|  |  | *Triaenodon obesus* | 85.7 | 22.2 |
|  |  | *Carcharhinus melanopterus* | 0.0 | 3.7 |
|  |  | *Nebrius ferrugineus* | 0.0 | 3.7 |
|  |  | *Stegostoma fasciatum* | 0.0 | 3.7 |
| **Rowley Shoals** | Reef | *Carcharhinus amblyrhynchos* | NA | 65.5 |
|  |  | *Triaenodon obesus* | NA | 34.5 |
| **Scott Reefs** | Reef | *Carcharhinus amblyrhynchos* | NA | 14.3 |
|  |  | *Triaenodon obesus* | NA | 85.7 |

**Appendix 3.** Model outputs for Negative Binomial Generalised Linear Models of combined MaxN of all predatory fish and shark species with predictor variables at Ashmore Reef. ‘Group’ represents size classes of mesopredatory fish (small, medium, and large) and sharks. The top ranked model is highlighted in bold font.

| **Habitat type** | **Model** | **K** | **logLik** | **AICc** | **ΔAICc** | **ΔAICc** | **D.E (%)** | **Null dev.** | **Resid. Dev.** |
| --- | --- | --- | --- | --- | --- | --- | --- | --- | --- |
| ***Reef habitat*** |  |  |  |  |  |  |  |  |  |
|  | **Year*Group+Depth** | **10** | **-202.53** | **427.92** | **0.00** | **0.69** | **0.56** | **200.75** | **87.39** |
|  | Year*Group+Depth+Coral | 11 | -202.18 | 429.84 | 1.92 | 0.26 | 0.57 | 200.98 | 86.78 |
|  | Year*Group | 9 | -206.57 | 433.44 | 5.52 | 0.04 | 0.52 | 180.96 | 86.98 |
|  | Depth | 3 | -236.34 | 478.97 | 51.05 | 0.00 | 0.06 | 102.08 | 95.78 |
|  | Depth*Year | 5 | -234.79 | 480.31 | 52.39 | 0.00 | 0.09 | 105.27 | 95.58 |
|  | Coral*Depth | 4 | -236.32 | 481.13 | 53.20 | 0.00 | 0.06 | 102.14 | 95.79 |
|  | Intercept | 2 | -239.39 | 482.92 | 55.00 | 0.00 | 0.00 | 96.32 | 96.32 |
|  | Complexity | 3 | -239.16 | 484.61 | 56.69 | 0.00 | 0.00 | 96.75 | 96.29 |
|  | Coral | 3 | -239.23 | 484.75 | 56.83 | 0.00 | 0.00 | 96.62 | 96.31 |
|  | Year | 3 | -239.35 | 484.98 | 57.06 | 0.00 | 0.00 | 96.41 | 96.32 |
|  | Coral*Year | 5 | -239.07 | 488.87 | 60.95 | 0.00 | 0.01 | 96.93 | 96.29 |
| ***Near-Reef habitat*** |  |  |  |  |  |  |  |  |  |
|  | **Year*Group** | **9** | **-316.82** | **653.11** | **1.71** | **0.26** | **0.30** | **204.06** | **143.01** |
|  | Year*Group+Depth+Coral | 11 | -313.60 | 651.40 | 0.00 | 0.60 | 0.33 | 211.29 | 141.24 |
|  | Year*Group+Depth | 10 | -316.24 | 654.30 | 2.89 | 0.14 | 0.31 | 205.03 | 142.49 |
|  | Year | 3 | -340.44 | 687.08 | 35.67 | 0.00 | 0.02 | 148.95 | 146.21 |
|  | Coral*Year | 5 | -338.33 | 687.14 | 35.73 | 0.00 | 0.05 | 153.32 | 146.18 |
|  | Intercept | 2 | -341.80 | 687.69 | 36.29 | 0.00 | 0.00 | 146.22 | 146.22 |
|  | Coral | 3 | -341.14 | 688.46 | 37.06 | 0.00 | 0.01 | 147.56 | 146.22 |
|  | Complexity | 3 | -341.59 | 689.36 | 37.96 | 0.00 | 0.00 | 146.64 | 146.22 |
|  | Depth | 3 | -341.65 | 689.50 | 38.09 | 0.00 | 0.00 | 146.50 | 146.21 |
|  | Coral*Depth | 4 | -340.92 | 690.16 | 38.76 | 0.00 | 0.01 | 148.00 | 146.23 |
|  | Depth*Year | 5 | -340.33 | 691.13 | 39.73 | 0.00 | 0.02 | 149.19 | 146.20 |
