# Supplementary material for: Protection from illegal fishing and shark recovery restructures mesopredatory fish communities on a coral reef
Source: Ecol Evol. 2019 Aug 20;9(18):10553–66. doi: 10.1002/ece3.5575 (PMC6787830; doi:10.1002/ece3.5575)
Supplement: Supplementary file 7 [file ECE3-9-10553-s007.docx]

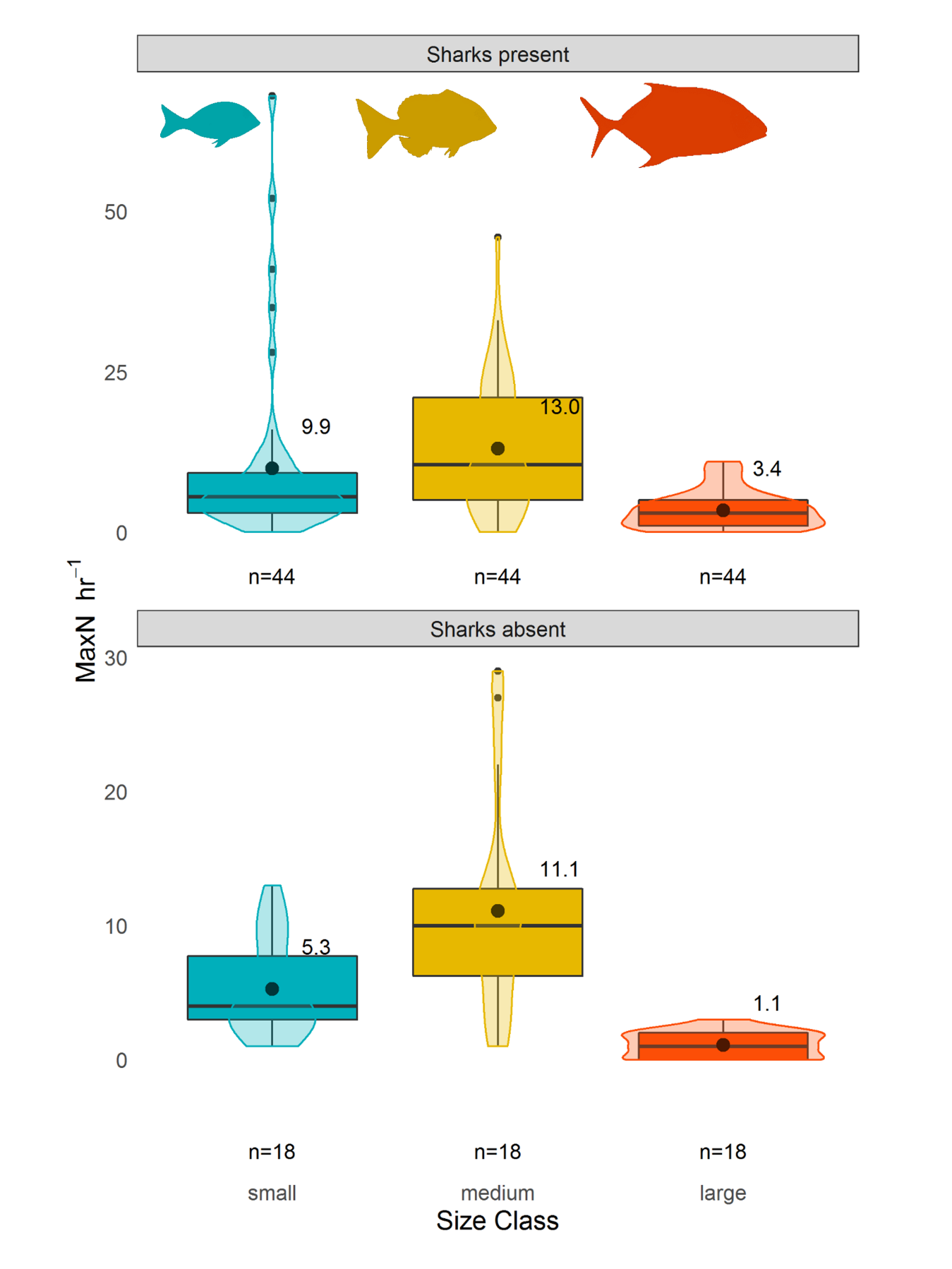


**Appendix 4.** Average abundance of mesopredatory fish in reef habitats in offshore north Western Australia, where sharks were present and absent on BRUVS deployments. Data include deployments from Ashmore (2004 and 2016), Scott Reefs (2016), and Rowley Shoals (2016).
